# Supplementary material for: How Do Gepotidacin and Zoliflodacin Stabilize DNA-Cleavage Complexes with Bacterial Type IIA Topoisomerases? 2. A Single Moving Metal Mechanism
Source: Int J Mol Sci. 2024 Dec 24;26(1):33. doi: 10.3390/ijms26010033 (PMC11720246; doi:10.3390/ijms26010033)
Supplement: Supplementary file 1 [file ijms-26-00033-s001.zip › supplementary-figs.pptx]

## Slide 1
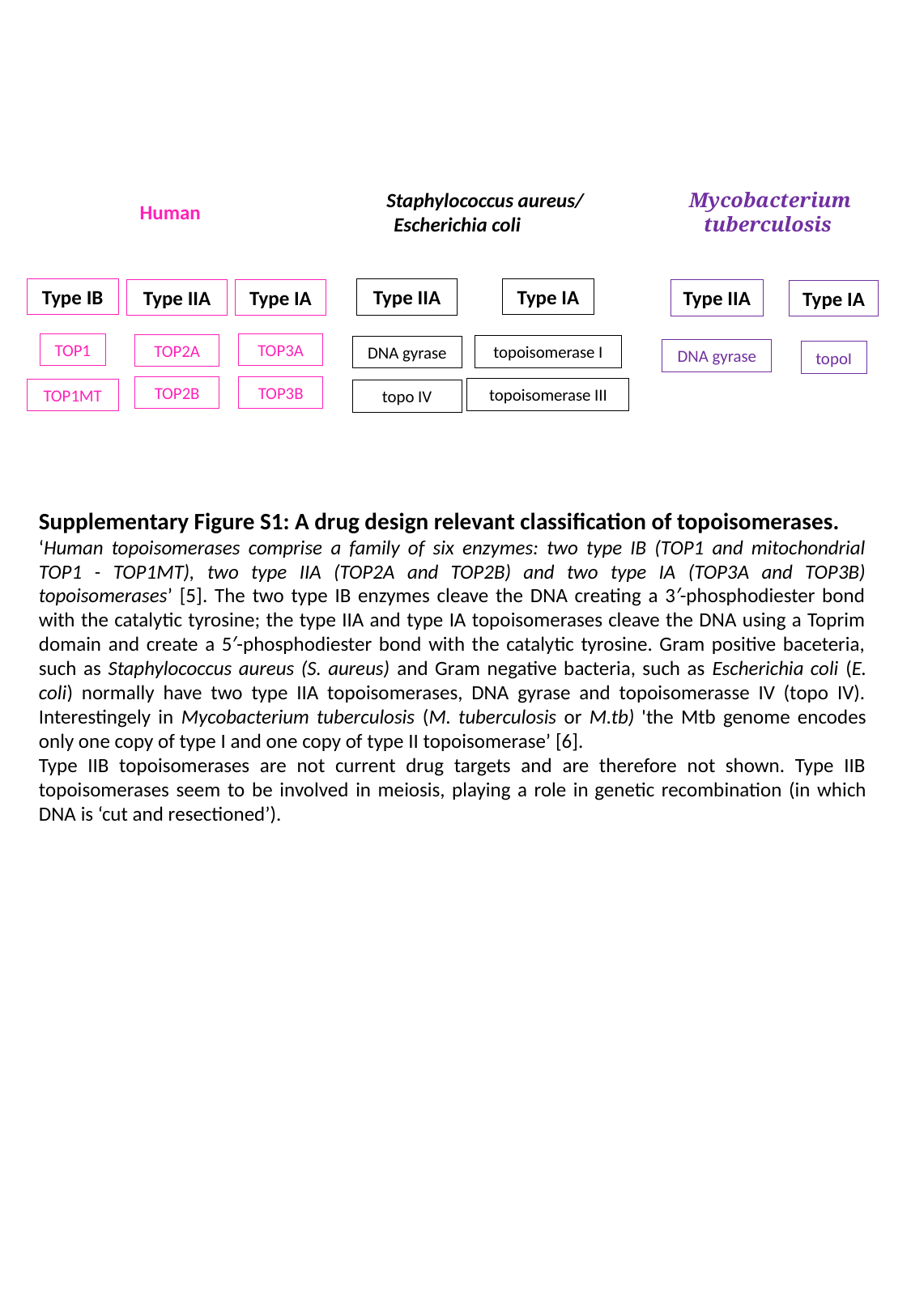

Staphylococcus aureus/ Escherichia coli
Mycobacterium tuberculosis
Human
Type IA
Type IB
Type IIA
Type IIA
Type IA
Type IIA
Type IA
TOP3A
TOP1
TOP2A
topoisomerase I
DNA gyrase
DNA gyrase
topoI
TOP2B
TOP3B
topoisomerase III
TOP1MT
topo IV
Supplementary Figure S1: A drug design relevant classification of topoisomerases.
‘Human topoisomerases comprise a family of six enzymes: two type IB (TOP1 and mitochondrial TOP1 - TOP1MT), two type IIA (TOP2A and TOP2B) and two type IA (TOP3A and TOP3B) topoisomerases’ [5]. The two type IB enzymes cleave the DNA creating a 3′-phosphodiester bond with the catalytic tyrosine; the type IIA and type IA topoisomerases cleave the DNA using a Toprim domain and create a 5′-phosphodiester bond with the catalytic tyrosine. Gram positive baceteria, such as Staphylococcus aureus (S. aureus) and Gram negative bacteria, such as Escherichia coli (E. coli) normally have two type IIA topoisomerases, DNA gyrase and topoisomerasse IV (topo IV). Interestingely in Mycobacterium tuberculosis (M. tuberculosis or M.tb) 'the Mtb genome encodes only one copy of type I and one copy of type II topoisomerase’ [6].
Type IIB topoisomerases are not current drug targets and are therefore not shown. Type IIB topoisomerases seem to be involved in meiosis, playing a role in genetic recombination (in which DNA is ‘cut and resectioned’).

## Slide 2
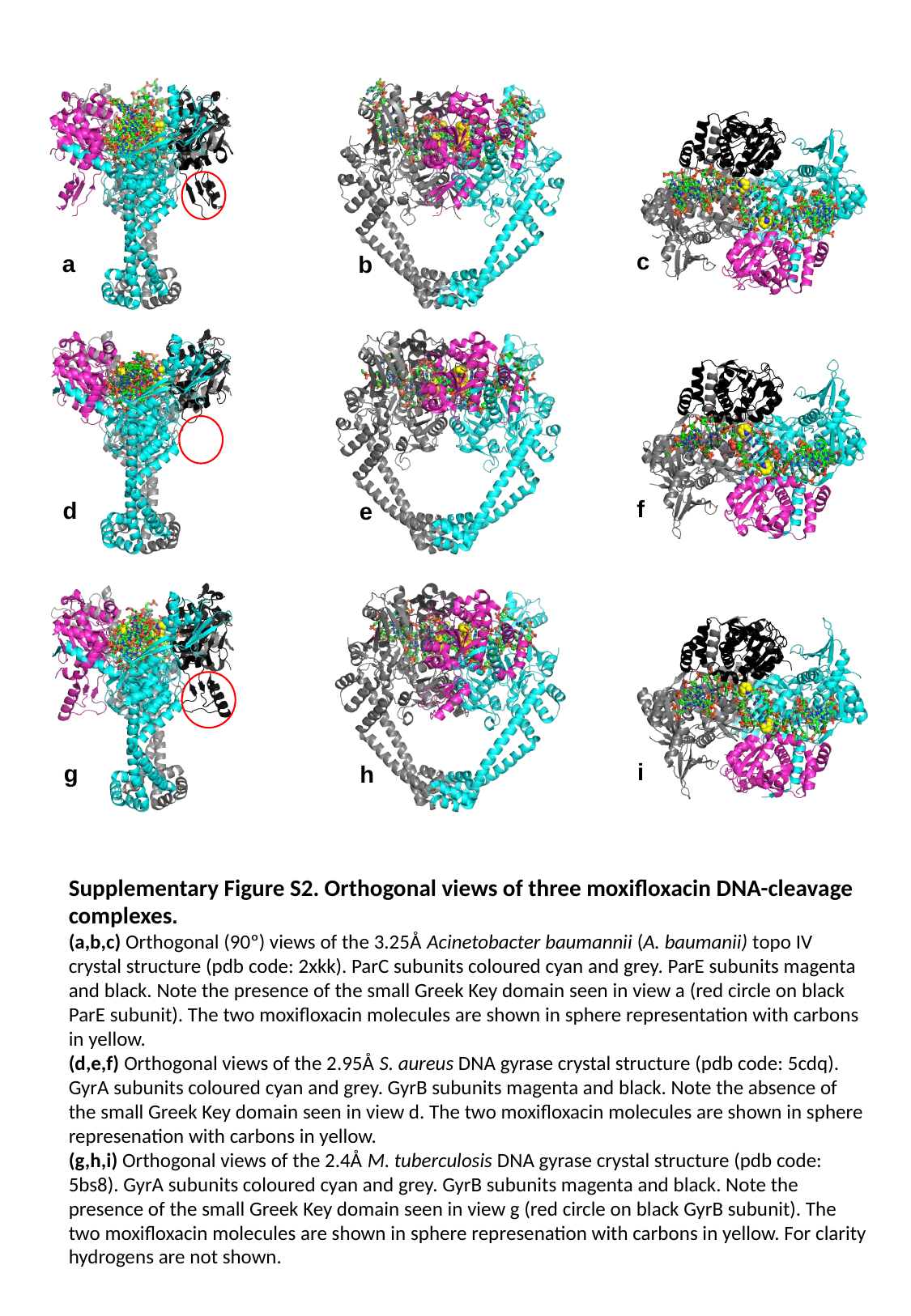

c
a
b
f
d
e
i
g
h
Supplementary Figure S2. Orthogonal views of three moxifloxacin DNA-cleavage complexes.
(a,b,c) Orthogonal (90º) views of the 3.25Å Acinetobacter baumannii (A. baumanii) topo IV crystal structure (pdb code: 2xkk). ParC subunits coloured cyan and grey. ParE subunits magenta and black. Note the presence of the small Greek Key domain seen in view a (red circle on black ParE subunit). The two moxifloxacin molecules are shown in sphere representation with carbons in yellow.
(d,e,f) Orthogonal views of the 2.95Å S. aureus DNA gyrase crystal structure (pdb code: 5cdq). GyrA subunits coloured cyan and grey. GyrB subunits magenta and black. Note the absence of the small Greek Key domain seen in view d. The two moxifloxacin molecules are shown in sphere represenation with carbons in yellow.
(g,h,i) Orthogonal views of the 2.4Å M. tuberculosis DNA gyrase crystal structure (pdb code: 5bs8). GyrA subunits coloured cyan and grey. GyrB subunits magenta and black. Note the presence of the small Greek Key domain seen in view g (red circle on black GyrB subunit). The two moxifloxacin molecules are shown in sphere represenation with carbons in yellow. For clarity hydrogens are not shown.

## Slide 3
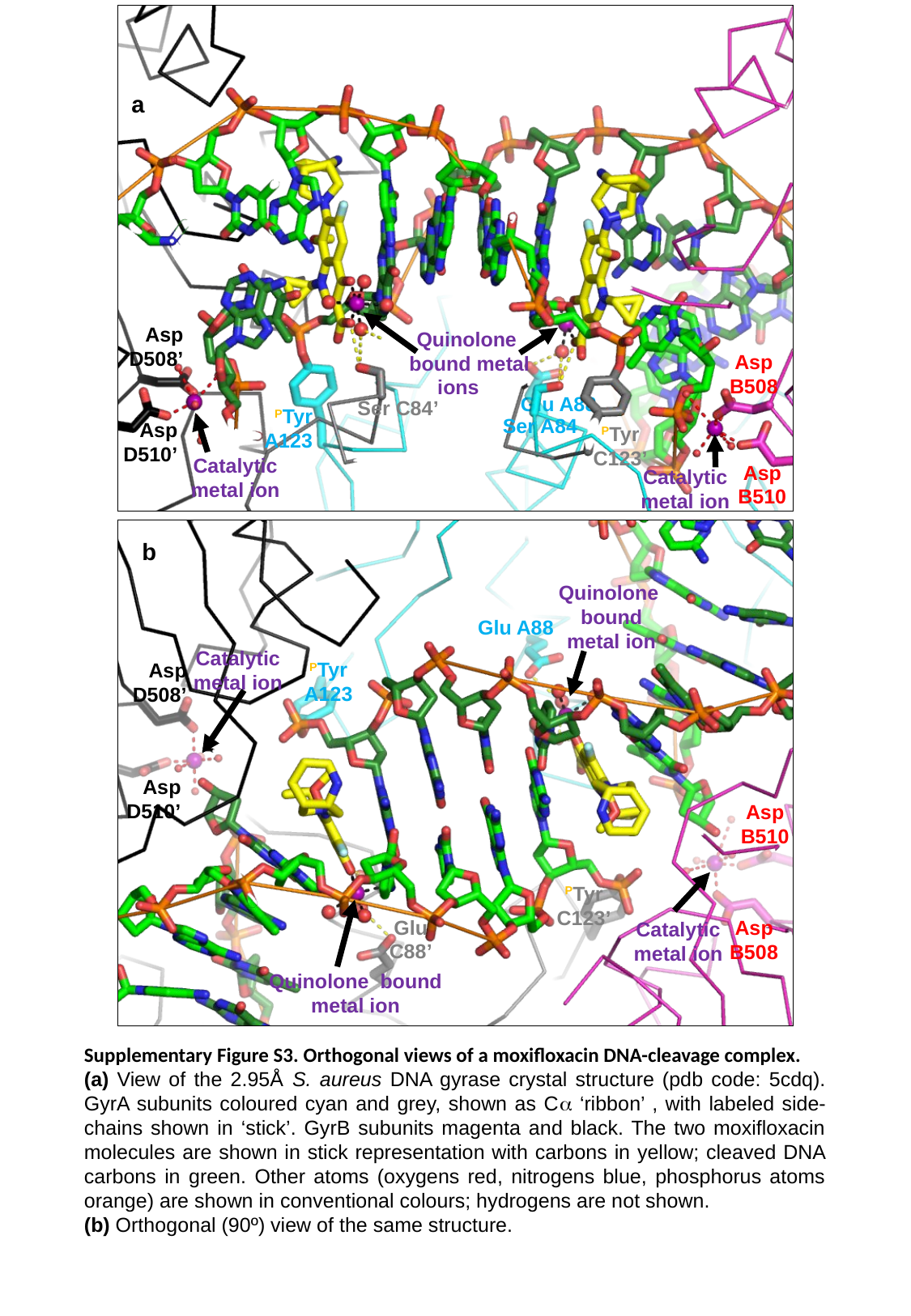

a
Asp D508’
Quinolone bound metal ions
Asp B508
Glu A88
Ser C84’
PTyr A123
Ser A84
Asp D510’
PTyr C123’
Catalytic metal ion
Asp B510
Catalytic metal ion
b
Quinolone bound metal ion
Glu A88
Catalytic metal ion
PTyr A123
Asp D508’
Asp D510’
Asp B510
PTyr C123’
Glu C88’
Asp B508
Catalytic metal ion
Quinolone bound metal ion
Supplementary Figure S3. Orthogonal views of a moxifloxacin DNA-cleavage complex.
(a) View of the 2.95Å S. aureus DNA gyrase crystal structure (pdb code: 5cdq). GyrA subunits coloured cyan and grey, shown as Ca ‘ribbon’ , with labeled side-chains shown in ‘stick’. GyrB subunits magenta and black. The two moxifloxacin molecules are shown in stick representation with carbons in yellow; cleaved DNA carbons in green. Other atoms (oxygens red, nitrogens blue, phosphorus atoms orange) are shown in conventional colours; hydrogens are not shown.
(b) Orthogonal (90º) view of the same structure.

## Slide 4
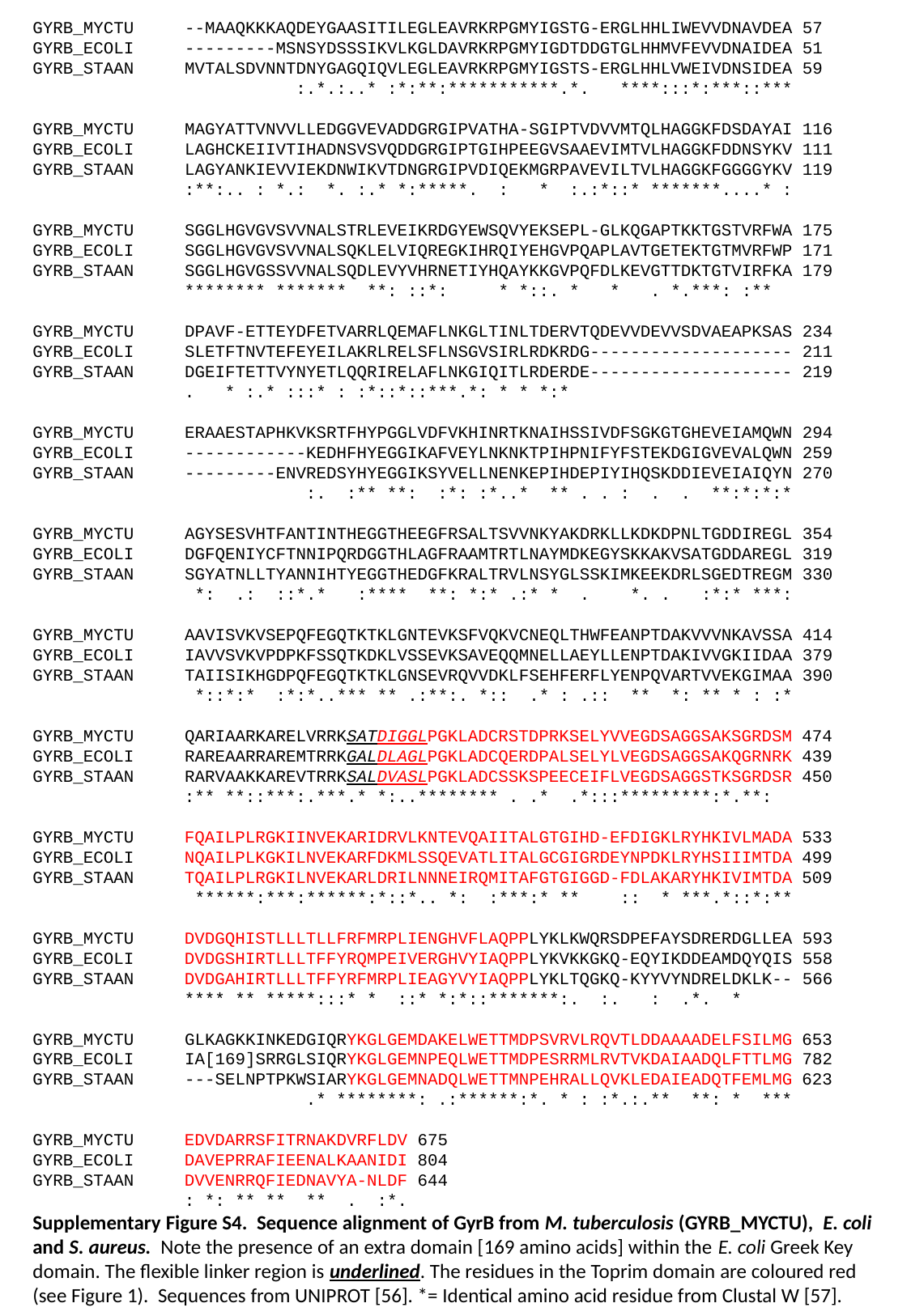

GYRB_MYCTU --MAAQKKKAQDEYGAASITILEGLEAVRKRPGMYIGSTG-ERGLHHLIWEVVDNAVDEA 57
GYRB_ECOLI ---------MSNSYDSSSIKVLKGLDAVRKRPGMYIGDTDDGTGLHHMVFEVVDNAIDEA 51
GYRB_STAAN MVTALSDVNNTDNYGAGQIQVLEGLEAVRKRPGMYIGSTS-ERGLHHLVWEIVDNSIDEA 59
 :.*.:..* :*:**:***********.*. ****:::*:***::***
GYRB_MYCTU MAGYATTVNVVLLEDGGVEVADDGRGIPVATHA-SGIPTVDVVMTQLHAGGKFDSDAYAI 116
GYRB_ECOLI LAGHCKEIIVTIHADNSVSVQDDGRGIPTGIHPEEGVSAAEVIMTVLHAGGKFDDNSYKV 111
GYRB_STAAN LAGYANKIEVVIEKDNWIKVTDNGRGIPVDIQEKMGRPAVEVILTVLHAGGKFGGGGYKV 119
 :**:.. : *.: *. :.* *:*****. : * :.:*::* *******....* :
GYRB_MYCTU SGGLHGVGVSVVNALSTRLEVEIKRDGYEWSQVYEKSEPL-GLKQGAPTKKTGSTVRFWA 175
GYRB_ECOLI SGGLHGVGVSVVNALSQKLELVIQREGKIHRQIYEHGVPQAPLAVTGETEKTGTMVRFWP 171
GYRB_STAAN SGGLHGVGSSVVNALSQDLEVYVHRNETIYHQAYKKGVPQFDLKEVGTTDKTGTVIRFKA 179
 ******** ******* **: ::*: * *::. * * . *.***: :**
GYRB_MYCTU DPAVF-ETTEYDFETVARRLQEMAFLNKGLTINLTDERVTQDEVVDEVVSDVAEAPKSAS 234
GYRB_ECOLI SLETFTNVTEFEYEILAKRLRELSFLNSGVSIRLRDKRDG-------------------- 211
GYRB_STAAN DGEIFTETTVYNYETLQQRIRELAFLNKGIQITLRDERDE-------------------- 219
 . * :.* :::* : :*::*::***.*: * * *:*
GYRB_MYCTU ERAAESTAPHKVKSRTFHYPGGLVDFVKHINRTKNAIHSSIVDFSGKGTGHEVEIAMQWN 294
GYRB_ECOLI ------------KEDHFHYEGGIKAFVEYLNKNKTPIHPNIFYFSTEKDGIGVEVALQWN 259
GYRB_STAAN ---------ENVREDSYHYEGGIKSYVELLNENKEPIHDEPIYIHQSKDDIEVEIAIQYN 270
 :. :** **: :*: :*..* ** . . : . . **:*:*:*
GYRB_MYCTU AGYSESVHTFANTINTHEGGTHEEGFRSALTSVVNKYAKDRKLLKDKDPNLTGDDIREGL 354
GYRB_ECOLI DGFQENIYCFTNNIPQRDGGTHLAGFRAAMTRTLNAYMDKEGYSKKAKVSATGDDAREGL 319
GYRB_STAAN SGYATNLLTYANNIHTYEGGTHEDGFKRALTRVLNSYGLSSKIMKEEKDRLSGEDTREGM 330
 *: .: ::*.* :**** **: *:* .:* * . *. . :*:* ***:
GYRB_MYCTU AAVISVKVSEPQFEGQTKTKLGNTEVKSFVQKVCNEQLTHWFEANPTDAKVVVNKAVSSA 414
GYRB_ECOLI IAVVSVKVPDPKFSSQTKDKLVSSEVKSAVEQQMNELLAEYLLENPTDAKIVVGKIIDAA 379
GYRB_STAAN TAIISIKHGDPQFEGQTKTKLGNSEVRQVVDKLFSEHFERFLYENPQVARTVVEKGIMAA 390
 *::*:* :*:*..*** ** .:**:. *:: .* : .:: ** *: ** * : :*
GYRB_MYCTU QARIAARKARELVRRKSATDIGGLPGKLADCRSTDPRKSELYVVEGDSAGGSAKSGRDSM 474
GYRB_ECOLI RAREAARRAREMTRRKGALDLAGLPGKLADCQERDPALSELYLVEGDSAGGSAKQGRNRK 439
GYRB_STAAN RARVAAKKAREVTRRKSALDVASLPGKLADCSSKSPEECEIFLVEGDSAGGSTKSGRDSR 450
 :** **::***:.***.* *:..******** . .* .*:::*********:*.**:
GYRB_MYCTU FQAILPLRGKIINVEKARIDRVLKNTEVQAIITALGTGIHD-EFDIGKLRYHKIVLMADA 533
GYRB_ECOLI NQAILPLKGKILNVEKARFDKMLSSQEVATLITALGCGIGRDEYNPDKLRYHSIIIMTDA 499
GYRB_STAAN TQAILPLRGKILNVEKARLDRILNNNEIRQMITAFGTGIGGD-FDLAKARYHKIVIMTDA 509
 ******:***:******:*::*.. *: :***:* ** :: * ***.*::*:**
GYRB_MYCTU DVDGQHISTLLLTLLFRFMRPLIENGHVFLAQPPLYKLKWQRSDPEFAYSDRERDGLLEA 593
GYRB_ECOLI DVDGSHIRTLLLTFFYRQMPEIVERGHVYIAQPPLYKVKKGKQ-EQYIKDDEAMDQYQIS 558
GYRB_STAAN DVDGAHIRTLLLTFFYRFMRPLIEAGYVYIAQPPLYKLTQGKQ-KYYVYNDRELDKLK-- 566
 **** ** *****:::* * ::* *:*::*******:. :. : .*. *
GYRB_MYCTU GLKAGKKINKEDGIQRYKGLGEMDAKELWETTMDPSVRVLRQVTLDDAAAADELFSILMG 653
GYRB_ECOLI IA[169]SRRGLSIQRYKGLGEMNPEQLWETTMDPESRRMLRVTVKDAIAADQLFTTLMG 782
GYRB_STAAN ---SELNPTPKWSIARYKGLGEMNADQLWETTMNPEHRALLQVKLEDAIEADQTFEMLMG 623
 .* ********: .:******:*. * : :*.:.** **: * ***
GYRB_MYCTU EDVDARRSFITRNAKDVRFLDV 675
GYRB_ECOLI DAVEPRRAFIEENALKAANIDI 804
GYRB_STAAN DVVENRRQFIEDNAVYA-NLDF 644
 : *: ** ** ** . :*.
Supplementary Figure S4. Sequence alignment of GyrB from M. tuberculosis (GYRB_MYCTU), E. coli and S. aureus. Note the presence of an extra domain [169 amino acids] within the E. coli Greek Key domain. The flexible linker region is underlined. The residues in the Toprim domain are coloured red (see Figure 1). Sequences from UNIPROT [56]. *= Identical amino acid residue from Clustal W [57].

## Slide 5
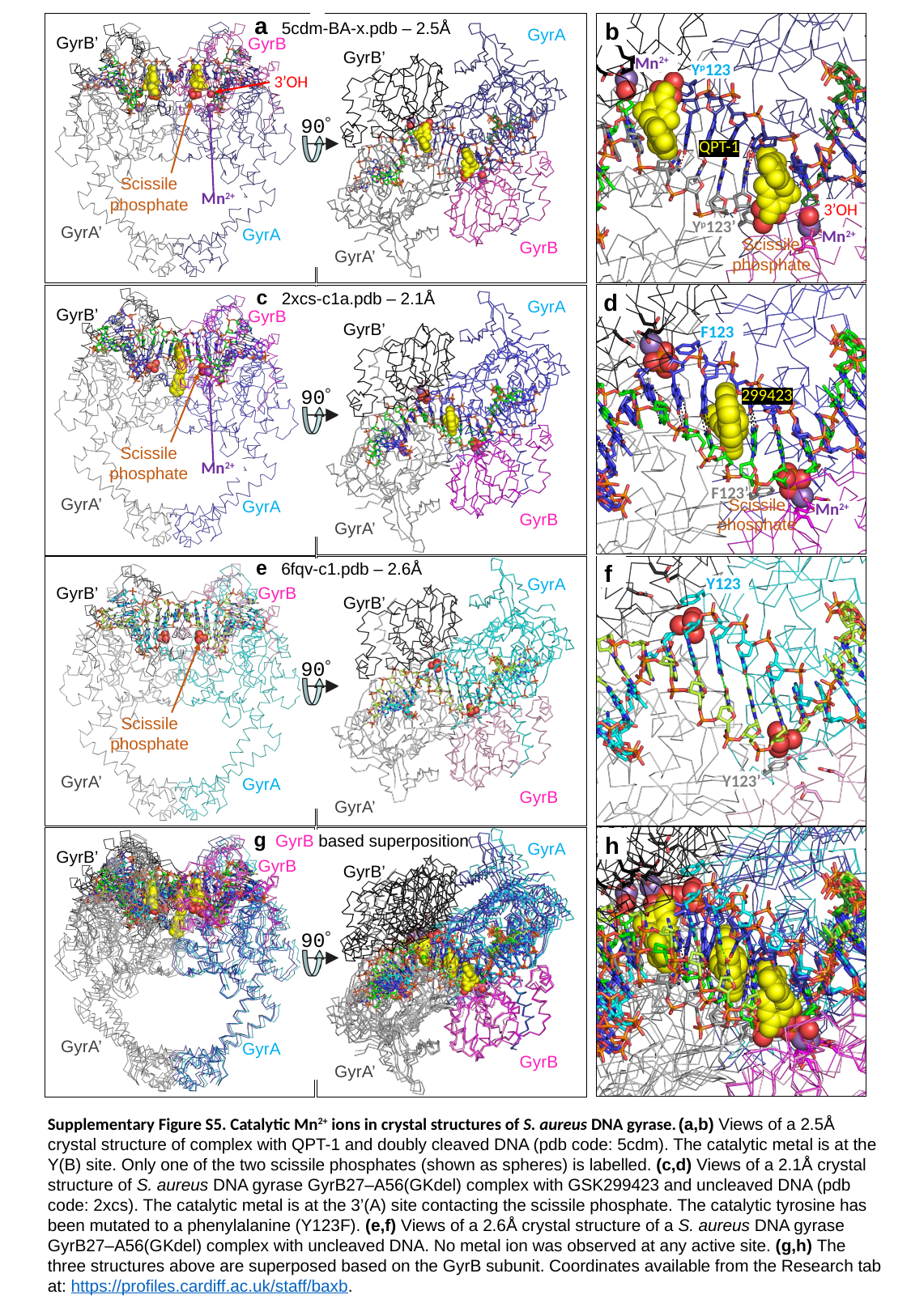

a 5cdm-BA-x.pdb – 2.5Å
b
90°
GyrA
GyrB’
GyrB
GyrB’
GyrA’
GyrA
GyrB
GyrA’
Mn2+
Yp123
3’OH
 QPT-1
Scissile phosphate
Mn2+
3’OH
Yp123’
Mn2+
Scissile phosphate
c 2xcs-c1a.pdb – 2.1Å
d
90°
GyrA
GyrB’
GyrB
GyrB’
GyrA’
GyrA
GyrB
GyrA’
F123
 299423
Scissile phosphate
Mn2+
F123’
Scissile phosphate
Mn2+
e 6fqv-c1.pdb – 2.6Å
f
90°
Y123
GyrA
GyrB’
GyrB
GyrB’
GyrA’
GyrA
GyrB
GyrA’
Scissile phosphate
Y123’
g GyrB based superposition
h
90°
GyrA
GyrB’
GyrB
GyrB’
GyrA’
GyrA
GyrB
GyrA’
Supplementary Figure S5. Catalytic Mn2+ ions in crystal structures of S. aureus DNA gyrase. (a,b) Views of a 2.5Å crystal structure of complex with QPT-1 and doubly cleaved DNA (pdb code: 5cdm). The catalytic metal is at the Y(B) site. Only one of the two scissile phosphates (shown as spheres) is labelled. (c,d) Views of a 2.1Å crystal structure of S. aureus DNA gyrase GyrB27–A56(GKdel) complex with GSK299423 and uncleaved DNA (pdb code: 2xcs). The catalytic metal is at the 3’(A) site contacting the scissile phosphate. The catalytic tyrosine has been mutated to a phenylalanine (Y123F). (e,f) Views of a 2.6Å crystal structure of a S. aureus DNA gyrase GyrB27–A56(GKdel) complex with uncleaved DNA. No metal ion was observed at any active site. (g,h) The three structures above are superposed based on the GyrB subunit. Coordinates available from the Research tab at: https://profiles.cardiff.ac.uk/staff/baxb.

## Slide 6
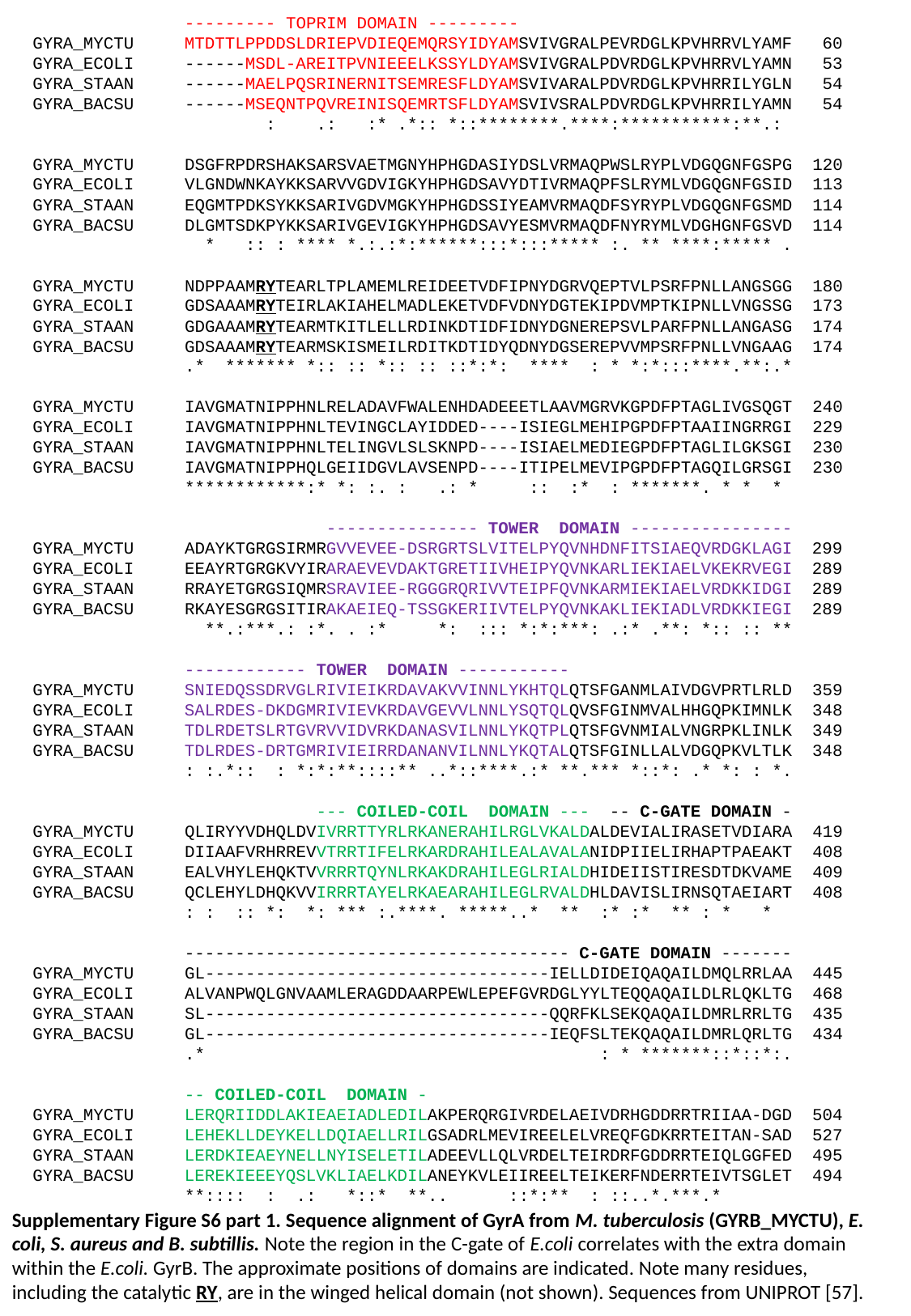

--------- TOPRIM DOMAIN ---------
GYRA_MYCTU MTDTTLPPDDSLDRIEPVDIEQEMQRSYIDYAMSVIVGRALPEVRDGLKPVHRRVLYAMF 60
GYRA_ECOLI ------MSDL-AREITPVNIEEELKSSYLDYAMSVIVGRALPDVRDGLKPVHRRVLYAMN 53
GYRA_STAAN ------MAELPQSRINERNITSEMRESFLDYAMSVIVARALPDVRDGLKPVHRRILYGLN 54
GYRA_BACSU ------MSEQNTPQVREINISQEMRTSFLDYAMSVIVSRALPDVRDGLKPVHRRILYAMN 54
 : .: :* .*:: *::********.****:***********:**.:
GYRA_MYCTU DSGFRPDRSHAKSARSVAETMGNYHPHGDASIYDSLVRMAQPWSLRYPLVDGQGNFGSPG 120
GYRA_ECOLI VLGNDWNKAYKKSARVVGDVIGKYHPHGDSAVYDTIVRMAQPFSLRYMLVDGQGNFGSID 113
GYRA_STAAN EQGMTPDKSYKKSARIVGDVMGKYHPHGDSSIYEAMVRMAQDFSYRYPLVDGQGNFGSMD 114
GYRA_BACSU DLGMTSDKPYKKSARIVGEVIGKYHPHGDSAVYESMVRMAQDFNYRYMLVDGHGNFGSVD 114
 * :: : **** *.:.:*:******:::*:::***** :. ** ****:***** .
GYRA_MYCTU NDPPAAMRYTEARLTPLAMEMLREIDEETVDFIPNYDGRVQEPTVLPSRFPNLLANGSGG 180
GYRA_ECOLI GDSAAAMRYTEIRLAKIAHELMADLEKETVDFVDNYDGTEKIPDVMPTKIPNLLVNGSSG 173
GYRA_STAAN GDGAAAMRYTEARMTKITLELLRDINKDTIDFIDNYDGNEREPSVLPARFPNLLANGASG 174
GYRA_BACSU GDSAAAMRYTEARMSKISMEILRDITKDTIDYQDNYDGSEREPVVMPSRFPNLLVNGAAG 174
 .* ******* *:: :: *:: :: ::*:*: **** : * *:*:::****.**:.*
GYRA_MYCTU IAVGMATNIPPHNLRELADAVFWALENHDADEEETLAAVMGRVKGPDFPTAGLIVGSQGT 240
GYRA_ECOLI IAVGMATNIPPHNLTEVINGCLAYIDDED----ISIEGLMEHIPGPDFPTAAIINGRRGI 229
GYRA_STAAN IAVGMATNIPPHNLTELINGVLSLSKNPD----ISIAELMEDIEGPDFPTAGLILGKSGI 230
GYRA_BACSU IAVGMATNIPPHQLGEIIDGVLAVSENPD----ITIPELMEVIPGPDFPTAGQILGRSGI 230
 ************:* *: :. : .: * :: :* : *******. * * *
 --------------- TOWER DOMAIN ----------------
GYRA_MYCTU ADAYKTGRGSIRMRGVVEVEE-DSRGRTSLVITELPYQVNHDNFITSIAEQVRDGKLAGI 299
GYRA_ECOLI EEAYRTGRGKVYIRARAEVEVDAKTGRETIIVHEIPYQVNKARLIEKIAELVKEKRVEGI 289
GYRA_STAAN RRAYETGRGSIQMRSRAVIEE-RGGGRQRIVVTEIPFQVNKARMIEKIAELVRDKKIDGI 289
GYRA_BACSU RKAYESGRGSITIRAKAEIEQ-TSSGKERIIVTELPYQVNKAKLIEKIADLVRDKKIEGI 289
 **.:***.: :*. . :* *: ::: *:*:***: .:* .**: *:: :: **
 ------------ TOWER DOMAIN -----------
GYRA_MYCTU SNIEDQSSDRVGLRIVIEIKRDAVAKVVINNLYKHTQLQTSFGANMLAIVDGVPRTLRLD 359
GYRA_ECOLI SALRDES-DKDGMRIVIEVKRDAVGEVVLNNLYSQTQLQVSFGINMVALHHGQPKIMNLK 348
GYRA_STAAN TDLRDETSLRTGVRVVIDVRKDANASVILNNLYKQTPLQTSFGVNMIALVNGRPKLINLK 349
GYRA_BACSU TDLRDES-DRTGMRIVIEIRRDANANVILNNLYKQTALQTSFGINLLALVDGQPKVLTLK 348
 : :.*:: : *:*:**::::** ..*::****.:* **.*** *::*: .* *: : *.
 --- COILED-COIL DOMAIN --- -- C-GATE DOMAIN -
GYRA_MYCTU QLIRYYVDHQLDVIVRRTTYRLRKANERAHILRGLVKALDALDEVIALIRASETVDIARA 419
GYRA_ECOLI DIIAAFVRHRREVVTRRTIFELRKARDRAHILEALAVALANIDPIIELIRHAPTPAEAKT 408
GYRA_STAAN EALVHYLEHQKTVVRRRTQYNLRKAKDRAHILEGLRIALDHIDEIISTIRESDTDKVAME 409
GYRA_BACSU QCLEHYLDHQKVVIRRRTAYELRKAEARAHILEGLRVALDHLDAVISLIRNSQTAEIART 408
 : : :: *: *: *** :.****. *****..* ** :* :* ** : * *
 -------------------------------------- C-GATE DOMAIN -------
GYRA_MYCTU GL----------------------------------IELLDIDEIQAQAILDMQLRRLAA 445
GYRA_ECOLI ALVANPWQLGNVAAMLERAGDDAARPEWLEPEFGVRDGLYYLTEQQAQAILDLRLQKLTG 468
GYRA_STAAN SL----------------------------------QQRFKLSEKQAQAILDMRLRRLTG 435
GYRA_BACSU GL----------------------------------IEQFSLTEKQAQAILDMRLQRLTG 434
 .* : * *******::*::*:.
 -- COILED-COIL DOMAIN -
GYRA_MYCTU LERQRIIDDLAKIEAEIADLEDILAKPERQRGIVRDELAEIVDRHGDDRRTRIIAA-DGD 504
GYRA_ECOLI LEHEKLLDEYKELLDQIAELLRILGSADRLMEVIREELELVREQFGDKRRTEITAN-SAD 527
GYRA_STAAN LERDKIEAEYNELLNYISELETILADEEVLLQLVRDELTEIRDRFGDDRRTEIQLGGFED 495
GYRA_BACSU LEREKIEEEYQSLVKLIAELKDILANEYKVLEIIREELTEIKERFNDERRTEIVTSGLET 494
 **:::: : .: *::* **.. ::*:** : ::..*.***.*
Supplementary Figure S6 part 1. Sequence alignment of GyrA from M. tuberculosis (GYRB_MYCTU), E. coli, S. aureus and B. subtillis. Note the region in the C-gate of E.coli correlates with the extra domain within the E.coli. GyrB. The approximate positions of domains are indicated. Note many residues, including the catalytic RY, are in the winged helical domain (not shown). Sequences from UNIPROT [57].

## Slide 7
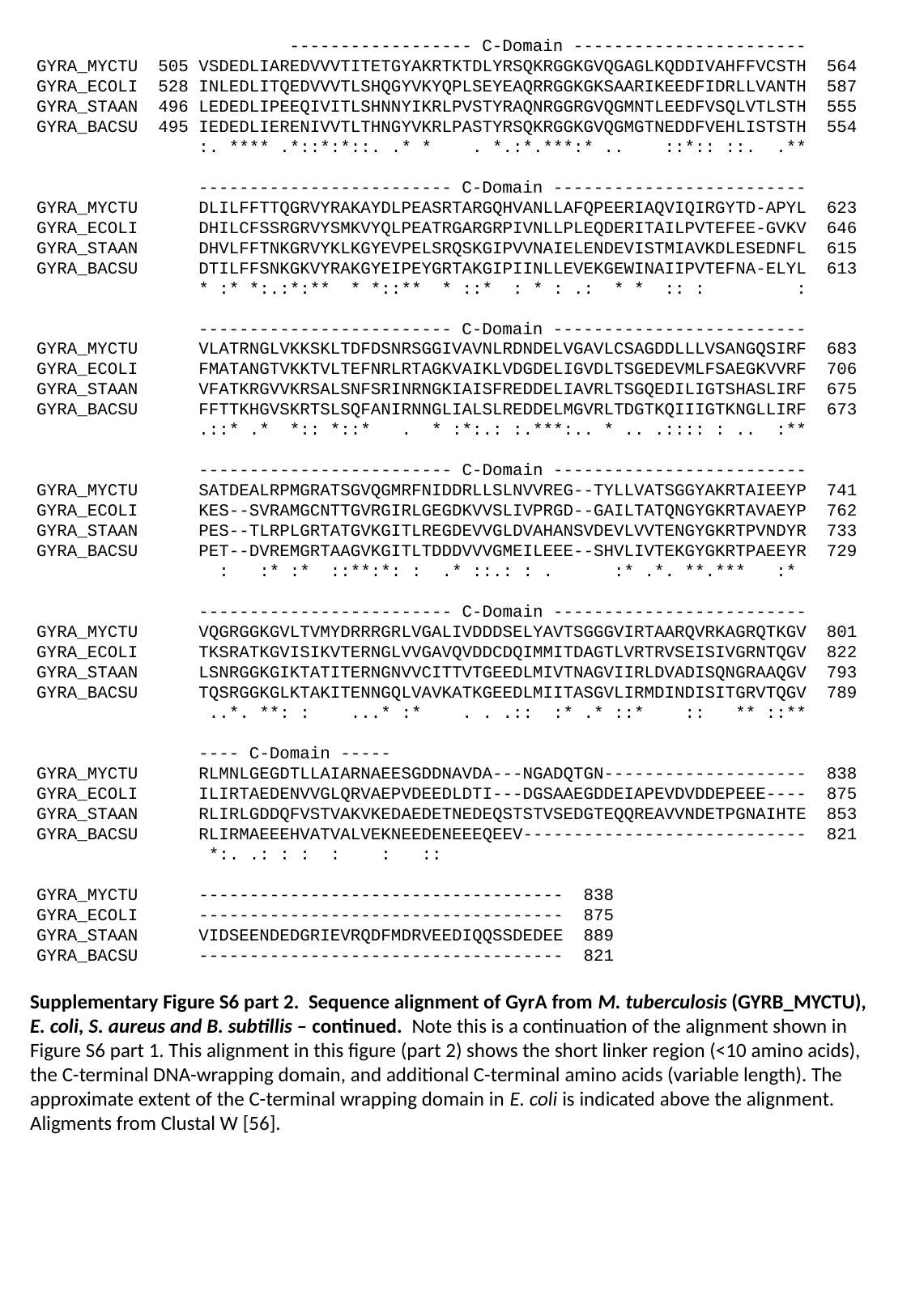

------------------ C-Domain -----------------------
GYRA_MYCTU 505 VSDEDLIAREDVVVTITETGYAKRTKTDLYRSQKRGGKGVQGAGLKQDDIVAHFFVCSTH 564
GYRA_ECOLI 528 INLEDLITQEDVVVTLSHQGYVKYQPLSEYEAQRRGGKGKSAARIKEEDFIDRLLVANTH 587
GYRA_STAAN 496 LEDEDLIPEEQIVITLSHNNYIKRLPVSTYRAQNRGGRGVQGMNTLEEDFVSQLVTLSTH 555
GYRA_BACSU 495 IEDEDLIERENIVVTLTHNGYVKRLPASTYRSQKRGGKGVQGMGTNEDDFVEHLISTSTH 554
 :. **** .*::*:*::. .* * . *.:*.***:* .. ::*:: ::. .**
 ------------------------- C-Domain -------------------------
GYRA_MYCTU DLILFFTTQGRVYRAKAYDLPEASRTARGQHVANLLAFQPEERIAQVIQIRGYTD-APYL 623
GYRA_ECOLI DHILCFSSRGRVYSMKVYQLPEATRGARGRPIVNLLPLEQDERITAILPVTEFEE-GVKV 646
GYRA_STAAN DHVLFFTNKGRVYKLKGYEVPELSRQSKGIPVVNAIELENDEVISTMIAVKDLESEDNFL 615
GYRA_BACSU DTILFFSNKGKVYRAKGYEIPEYGRTAKGIPIINLLEVEKGEWINAIIPVTEFNA-ELYL 613
 * :* *:.:*:** * *::** * ::* : * : .: * * :: : :
 ------------------------- C-Domain -------------------------
GYRA_MYCTU VLATRNGLVKKSKLTDFDSNRSGGIVAVNLRDNDELVGAVLCSAGDDLLLVSANGQSIRF 683
GYRA_ECOLI FMATANGTVKKTVLTEFNRLRTAGKVAIKLVDGDELIGVDLTSGEDEVMLFSAEGKVVRF 706
GYRA_STAAN VFATKRGVVKRSALSNFSRINRNGKIAISFREDDELIAVRLTSGQEDILIGTSHASLIRF 675
GYRA_BACSU FFTTKHGVSKRTSLSQFANIRNNGLIALSLREDDELMGVRLTDGTKQIIIGTKNGLLIRF 673
 .::* .* *:: *::* . * :*:.: :.***:.. * .. .:::: : .. :**
 ------------------------- C-Domain -------------------------
GYRA_MYCTU SATDEALRPMGRATSGVQGMRFNIDDRLLSLNVVREG--TYLLVATSGGYAKRTAIEEYP 741
GYRA_ECOLI KES--SVRAMGCNTTGVRGIRLGEGDKVVSLIVPRGD--GAILTATQNGYGKRTAVAEYP 762
GYRA_STAAN PES--TLRPLGRTATGVKGITLREGDEVVGLDVAHANSVDEVLVVTENGYGKRTPVNDYR 733
GYRA_BACSU PET--DVREMGRTAAGVKGITLTDDDVVVGMEILEEE--SHVLIVTEKGYGKRTPAEEYR 729
 : :* :* ::**:*: : .* ::.: : . :* .*. **.*** :*
 ------------------------- C-Domain -------------------------
GYRA_MYCTU VQGRGGKGVLTVMYDRRRGRLVGALIVDDDSELYAVTSGGGVIRTAARQVRKAGRQTKGV 801
GYRA_ECOLI TKSRATKGVISIKVTERNGLVVGAVQVDDCDQIMMITDAGTLVRTRVSEISIVGRNTQGV 822
GYRA_STAAN LSNRGGKGIKTATITERNGNVVCITTVTGEEDLMIVTNAGVIIRLDVADISQNGRAAQGV 793
GYRA_BACSU TQSRGGKGLKTAKITENNGQLVAVKATKGEEDLMIITASGVLIRMDINDISITGRVTQGV 789
 ..*. **: : ...* :* . . .:: :* .* ::* :: ** ::**
 ---- C-Domain -----
GYRA_MYCTU RLMNLGEGDTLLAIARNAEESGDDNAVDA---NGADQTGN-------------------- 838
GYRA_ECOLI ILIRTAEDENVVGLQRVAEPVDEEDLDTI---DGSAAEGDDEIAPEVDVDDEPEEE---- 875
GYRA_STAAN RLIRLGDDQFVSTVAKVKEDAEDETNEDEQSTSTVSEDGTEQQREAVVNDETPGNAIHTE 853
GYRA_BACSU RLIRMAEEEHVATVALVEKNEEDENEEEQEEV---------------------------- 821
 *:. .: : : : : ::
GYRA_MYCTU ------------------------------------ 838
GYRA_ECOLI ------------------------------------ 875
GYRA_STAAN VIDSEENDEDGRIEVRQDFMDRVEEDIQQSSDEDEE 889
GYRA_BACSU ------------------------------------ 821
Supplementary Figure S6 part 2. Sequence alignment of GyrA from M. tuberculosis (GYRB_MYCTU), E. coli, S. aureus and B. subtillis – continued. Note this is a continuation of the alignment shown in Figure S6 part 1. This alignment in this figure (part 2) shows the short linker region (<10 amino acids), the C-terminal DNA-wrapping domain, and additional C-terminal amino acids (variable length). The approximate extent of the C-terminal wrapping domain in E. coli is indicated above the alignment. Aligments from Clustal W [56].

## Slide 8
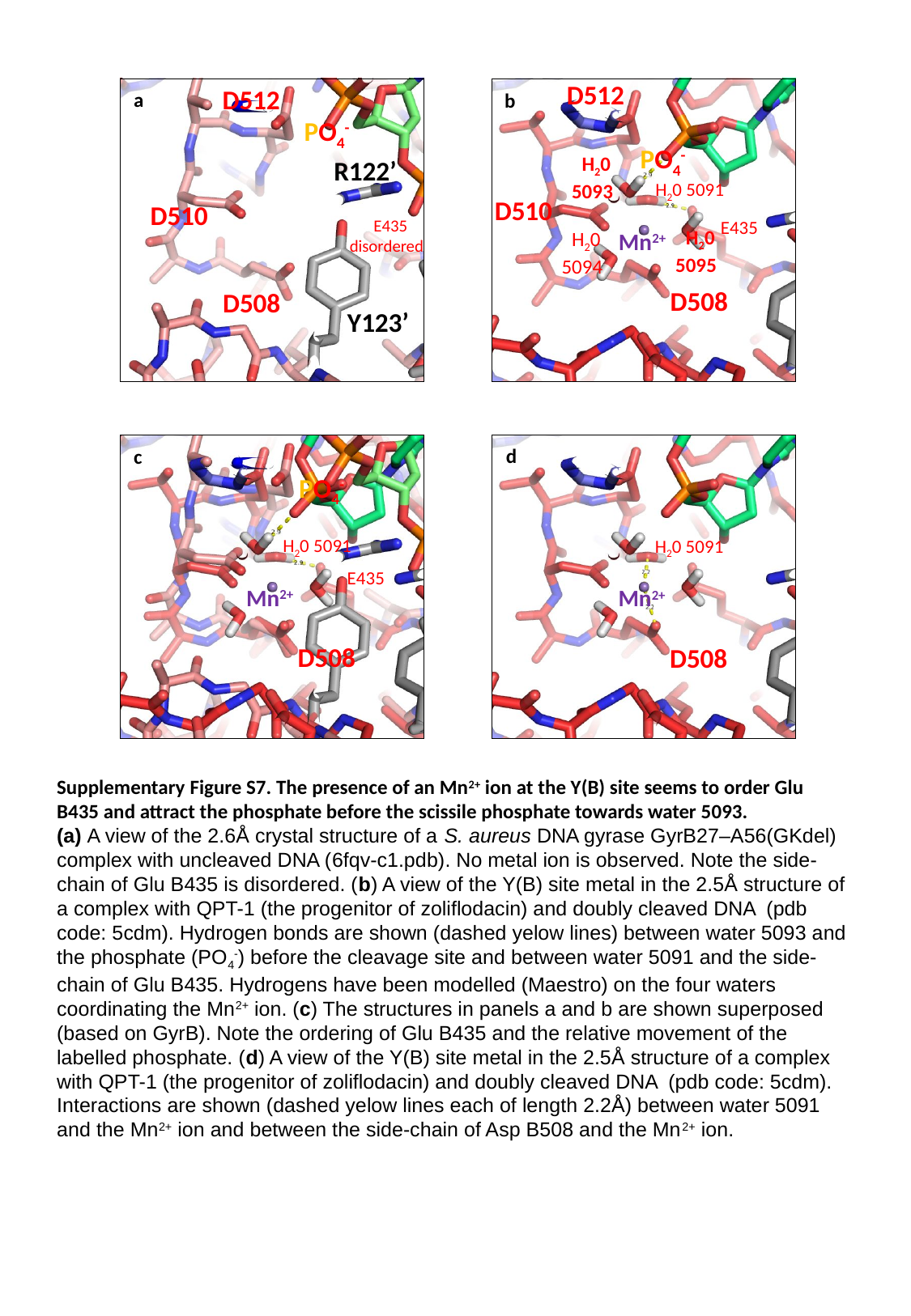

D512
 D512
a
b
 PO4-
 PO4-
 H20 5093
 R122’
 H20 5091
 D510
 D510
 E435
disordered
 E435
 Mn2+
 H20 5095
 H20 5094
 D508
 D508
 Y123’
c
d
 PO4-
 H20 5091
 H20 5091
 E435
 Mn2+
 Mn2+
 D508
 D508
Supplementary Figure S7. The presence of an Mn2+ ion at the Y(B) site seems to order Glu B435 and attract the phosphate before the scissile phosphate towards water 5093.
(a) A view of the 2.6Å crystal structure of a S. aureus DNA gyrase GyrB27–A56(GKdel) complex with uncleaved DNA (6fqv-c1.pdb). No metal ion is observed. Note the side-chain of Glu B435 is disordered. (b) A view of the Y(B) site metal in the 2.5Å structure of a complex with QPT-1 (the progenitor of zoliflodacin) and doubly cleaved DNA (pdb code: 5cdm). Hydrogen bonds are shown (dashed yelow lines) between water 5093 and the phosphate (PO4-) before the cleavage site and between water 5091 and the side-chain of Glu B435. Hydrogens have been modelled (Maestro) on the four waters coordinating the Mn2+ ion. (c) The structures in panels a and b are shown superposed (based on GyrB). Note the ordering of Glu B435 and the relative movement of the labelled phosphate. (d) A view of the Y(B) site metal in the 2.5Å structure of a complex with QPT-1 (the progenitor of zoliflodacin) and doubly cleaved DNA (pdb code: 5cdm). Interactions are shown (dashed yelow lines each of length 2.2Å) between water 5091 and the Mn2+ ion and between the side-chain of Asp B508 and the Mn2+ ion.

## Slide 9
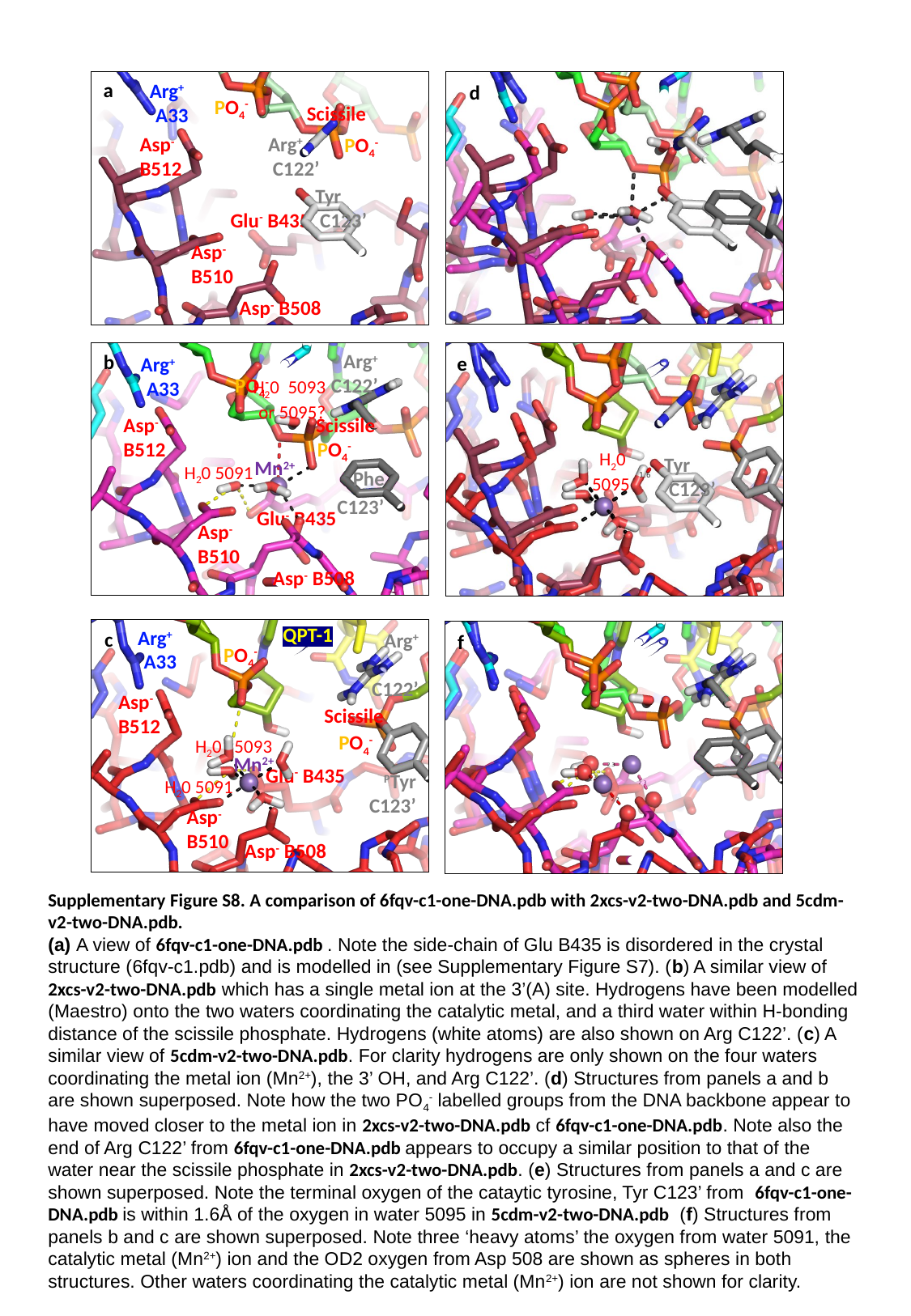

a
Arg+
A33
d
 PO4-
Scissile
Arg+
C122’
Asp- B512
 PO4-
Tyr
C123’
Glu- B435
Asp- B510
Asp- B508
Arg+
C122’
b
e
Arg+
A33
 PO4-
 H20 5093 or 5095?
Scissile
Asp- B512
 PO4-
 H20
5095
Tyr
C123’
 Mn2+
 H20 5091
Phe
C123’
Glu- B435
Asp- B510
Asp- B508
QPT-1
Arg+
A33
c
Arg+
C122’
f
 PO4-
Asp- B512
Scissile
 PO4-
 H20 5093
 Mn2+
Glu- B435
PTyr
C123’
 H20 5091
Asp- B510
Asp- B508
Supplementary Figure S8. A comparison of 6fqv-c1-one-DNA.pdb with 2xcs-v2-two-DNA.pdb and 5cdm-v2-two-DNA.pdb.
(a) A view of 6fqv-c1-one-DNA.pdb . Note the side-chain of Glu B435 is disordered in the crystal structure (6fqv-c1.pdb) and is modelled in (see Supplementary Figure S7). (b) A similar view of 2xcs-v2-two-DNA.pdb which has a single metal ion at the 3’(A) site. Hydrogens have been modelled (Maestro) onto the two waters coordinating the catalytic metal, and a third water within H-bonding distance of the scissile phosphate. Hydrogens (white atoms) are also shown on Arg C122’. (c) A similar view of 5cdm-v2-two-DNA.pdb. For clarity hydrogens are only shown on the four waters coordinating the metal ion (Mn2+), the 3’ OH, and Arg C122’. (d) Structures from panels a and b are shown superposed. Note how the two PO4- labelled groups from the DNA backbone appear to have moved closer to the metal ion in 2xcs-v2-two-DNA.pdb cf 6fqv-c1-one-DNA.pdb. Note also the end of Arg C122’ from 6fqv-c1-one-DNA.pdb appears to occupy a similar position to that of the water near the scissile phosphate in 2xcs-v2-two-DNA.pdb. (e) Structures from panels a and c are shown superposed. Note the terminal oxygen of the cataytic tyrosine, Tyr C123’ from 6fqv-c1-one-DNA.pdb is within 1.6Å of the oxygen in water 5095 in 5cdm-v2-two-DNA.pdb (f) Structures from panels b and c are shown superposed. Note three ‘heavy atoms’ the oxygen from water 5091, the catalytic metal (Mn2+) ion and the OD2 oxygen from Asp 508 are shown as spheres in both structures. Other waters coordinating the catalytic metal (Mn2+) ion are not shown for clarity.

## Slide 10
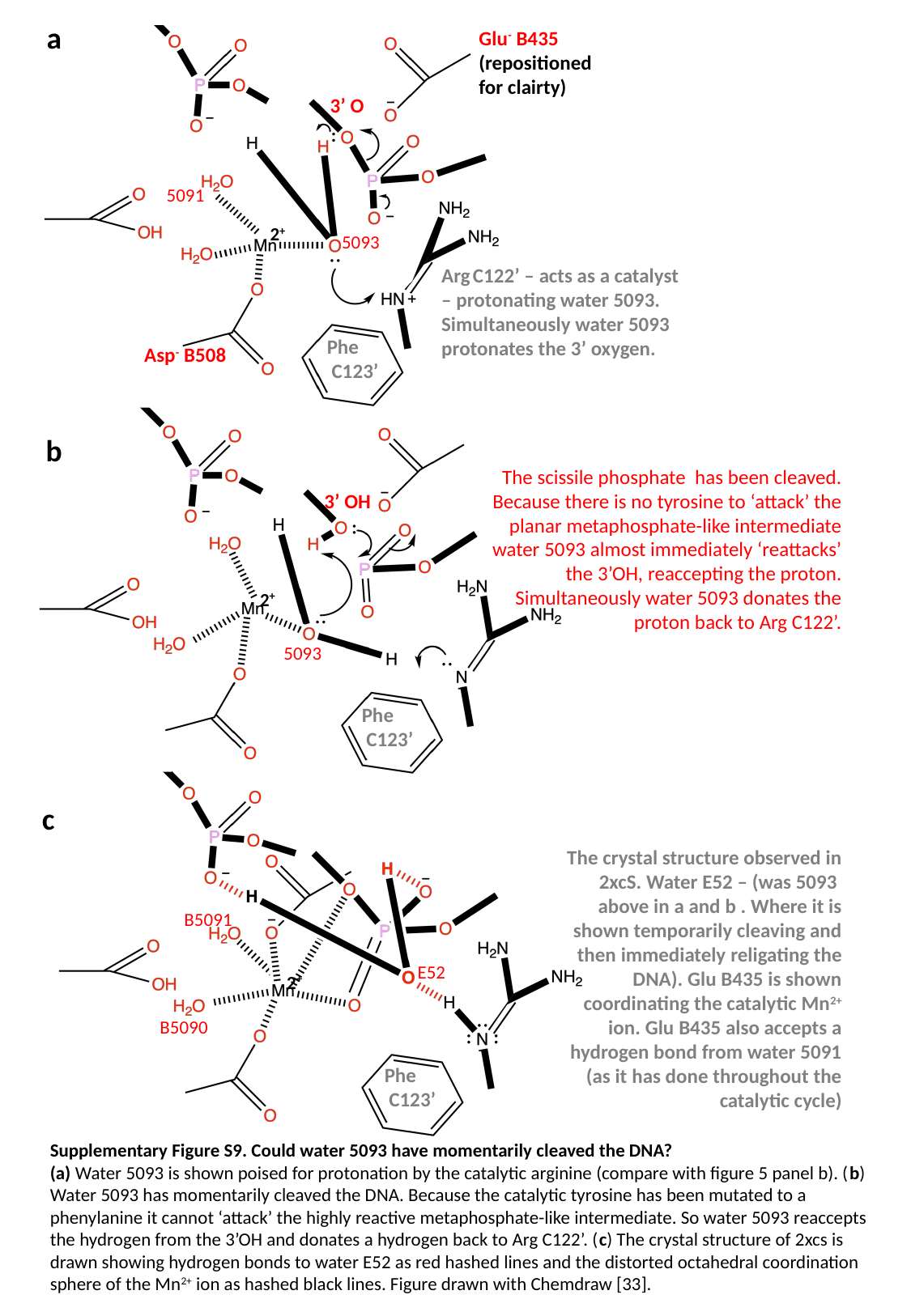

a
Glu- B435
(repositioned for clairty)
3’ O
 5091
2+
 5093
Arg C122’ – acts as a catalyst – protonating water 5093. Simultaneously water 5093 protonates the 3’ oxygen.
Phe
C123’
Asp- B508
b
The scissile phosphate has been cleaved. Because there is no tyrosine to ‘attack’ the planar metaphosphate-like intermediate water 5093 almost immediately ‘reattacks’ the 3’OH, reaccepting the proton. Simultaneously water 5093 donates the proton back to Arg C122’.
3’ OH
2+
 5093
Phe
C123’
c
The crystal structure observed in 2xcS. Water E52 – (was 5093 above in a and b . Where it is shown temporarily cleaving and then immediately religating the DNA). Glu B435 is shown coordinating the catalytic Mn2+ ion. Glu B435 also accepts a hydrogen bond from water 5091 (as it has done throughout the catalytic cycle)
 B5091
 E52
2+
 B5090
Phe
C123’
Supplementary Figure S9. Could water 5093 have momentarily cleaved the DNA?
(a) Water 5093 is shown poised for protonation by the catalytic arginine (compare with figure 5 panel b). (b) Water 5093 has momentarily cleaved the DNA. Because the catalytic tyrosine has been mutated to a phenylanine it cannot ‘attack’ the highly reactive metaphosphate-like intermediate. So water 5093 reaccepts the hydrogen from the 3’OH and donates a hydrogen back to Arg C122’. (c) The crystal structure of 2xcs is drawn showing hydrogen bonds to water E52 as red hashed lines and the distorted octahedral coordination sphere of the Mn2+ ion as hashed black lines. Figure drawn with Chemdraw [33].
