# Supplementary figures and images for: How Do Gepotidacin and Zoliflodacin Stabilize DNA-Cleavage Complexes with Bacterial Type IIA Topoisomerases? 2. A Single Moving Metal Mechanism
Source: Int J Mol Sci. 2024 Dec 24;26(1):33. doi: 10.3390/ijms26010033 (PMC11720246; doi:10.3390/ijms26010033)

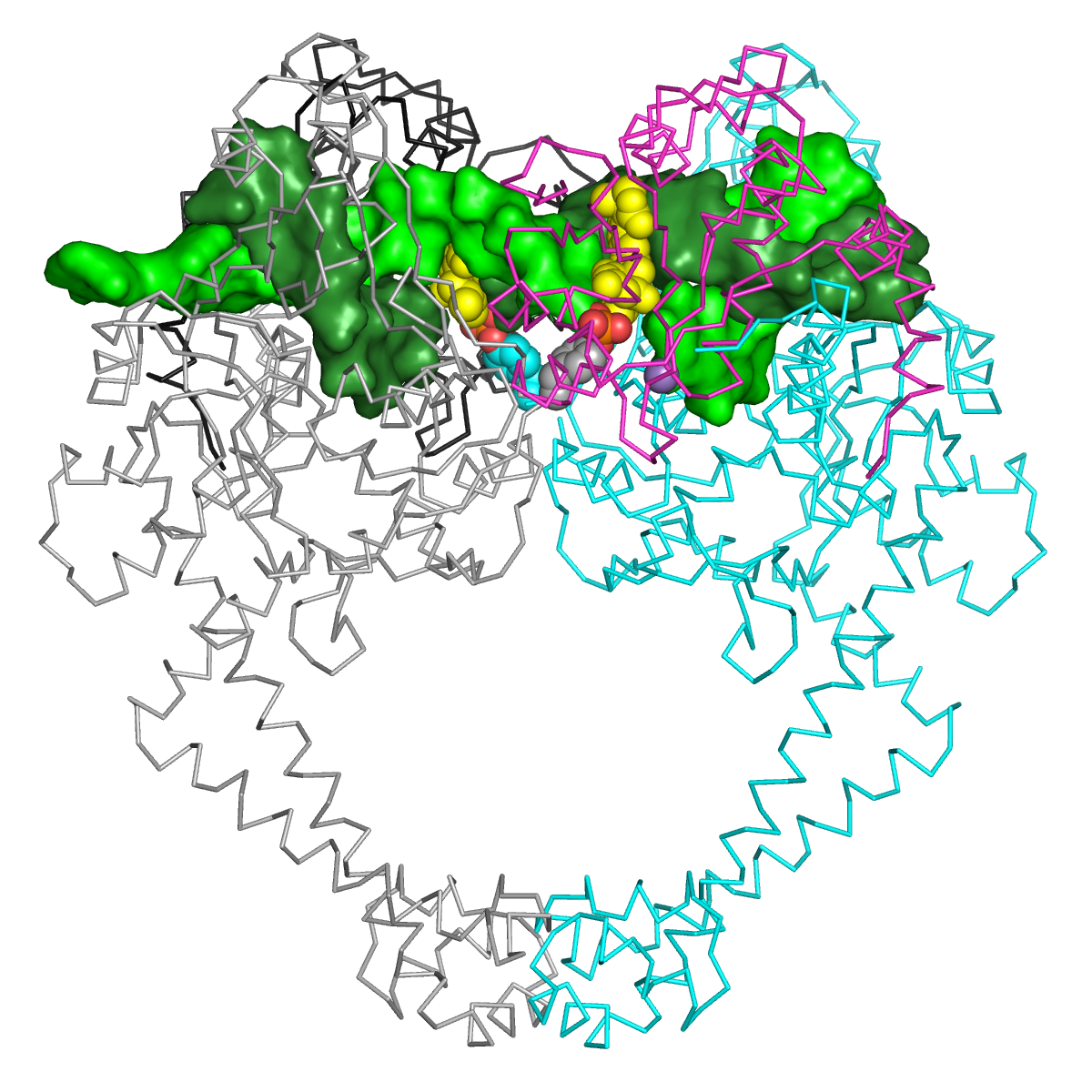

Supplement: Supplementary file 1 [file ijms-26-00033-s001.zip › fig1d.png]
